# Food Safety Knowledge, Attitudes, and Practices among Vegetable Handlers in Bangladesh

**DOI:** 10.1016/j.jfp.2024.100428

**Published:** 2025-01-02

**Authors:** Ismat Ara Begum, Mohammad Jahangir Alam, Bhavani Shankar, Tamanna Mastura, Gregory Cooper, Karl Rich, Panam Parikh, Nazmun N. Ratna, Suneetha Kadiyala

**Affiliations:** 1 Department of Agricultural Economics, Bangladesh Agricultural University, Mymensingh, Bangladesh; 2 Department of Agribusiness and Marketing, Bangladesh Agricultural University, Mymensingh, Bangladesh; 3 Institute for Sustainable Food & Department of Geography, The University of Sheffield, Sheffield, United Kingdom; 4 Ferguson College of Agriculture, Oklahoma State University, USA; 5 Nutrition for Impact, 30 Cecil Street, Singapore 049712, Singapore; 6 Faculty of Agribusiness & Commerce, Lincoln University, New Zealand; 7 London School of Hygiene and Tropical Medicine (LSHTM), London, United Kingdom

**Keywords:** Attitudes, Bangladesh, Food Safety, Handlers, Horticultural or Vegetable Value Chain, Knowledge, Practices

## Abstract

This study delves into the Knowledge, Attitudes, and Practices of vegetable handlers in Jashore Sadar, Bangladesh, specifically focusing on the crucial significance of food safety across the entire farm-to-fork process. Integrating quantitative and qualitative data provides a multifaceted depiction of this pivotal component within the food supply chain. Ten focus group discussions were conducted, and in total, 50 vegetable handlers were interviewed separately. In the survey, there were 17 questions on food safety knowledge, 16 on food safety attitudes, and 17 on food safety-related practices followed by vegetable handlers. We found that while approximately 60% of handlers had good knowledge about food safety, only about one-quarter of the interviewed vegetable handlers had good attitudes and practices toward food safety. The attitudes and practices of individuals involved in handling vegetables demonstrate a complex range of variations shaped by factors such as educational background, prior experience, and availability of food safety training. Concerns regarding the rising expenses linked to adherence to food safety regulations and a perceived lack of accountability among those involved were also noted. The interviewed wholesaler group reported that approximately BDT 100–150 (USD 0.91–1.37^1^)/maund^1^ was incurred in maintaining proper safety practices, while the interviewed retailer group said costs would increase by BDT 20–40 (USD 0.18–0.36)/maund if they followed proper handling practices. It is important to note that vegetable handlers recognize the favorable economic consequences of implementing appropriate food safety measures. This study highlights the significance of implementing focused educational and policy interventions to address knowledge disparities and encourage the adoption of food safety procedures among individuals involved in handling vegetables.

## Highlights

• This study examines food safety-related knowledge, attitudes, and practices of vegetable handlers.
• The mixed-methods approach provides a detailed view of food safety within the supply chain.
• In the study areas, 50 vegetable handlers were interviewed, and 10 focus group discussions were conducted.
• Among the respondents, 60% of handlers had good knowledge, but few demonstrated good attitudes and practices.
• Economic benefits motivate safety practices, but costs remain a significant concern.

The United Nations Sustainable Development Goals aim to transform our world. They are a call to end global hunger, malnutrition, and poverty by 2030, for which ensuring food safety is a must. Unsafe food is a global public health issue resulting in 0.42 million deaths and the loss of 33 million healthy life years annually (WHO 2015). Low-income countries (LICs) and Lower-Middle-Income countries (LMICs) bear a large part of this burden due to their reliance on informal markets with poor infrastructure and weak food safety regulations (FAO, 2020).

Bangladesh is one of the most densely populated countries in the world, with about 70% of the country’s land area feeding a population of over 170 million, of which more than three-quarters are farming households (Bangladesh Bureau of Statistics, 2020, Goosen et al., 2018). Bangladesh produced over 26.7 million tons of vegetables in 2018–2019 (DAE, 2019), the majority of which were sold in traditional open-air (“*wet*”) markets. As in many other LMICs (Gómez & Ricketts, 2013), these conventional markets often interface between long agricultural value chains and lower-income consumers. Inconsistent food safety practices and poor infrastructure at any point in the agricultural value chain can impose a high risk of food contamination and a consequent risk of food-borne diseases. One of the food groups most often implicated in food-borne diseases is fresh produce such as vegetables and fruits (Parikh et al., 2022).

Vegetables can be contaminated during production, transportation, packaging, storage, sale, or by inadequate cooking practices (Degaga et al., 2022). Studies from Bangladesh and other low and middle-income countries (LMICs) point to various sources of vegetable contamination, which increase the dietary exposure risk for farmers and the general population. Vegetable cultivators in Bangladesh are reported to have unacceptably high pesticide use, applying an average of 3.4 kg per hectare per growing season, (Dasgupta & Meisner, 2005), while using other chemicals, such as food contact materials and cleaning agents, that act as contaminants in vegetable production (Rather et al., 2017). This level, nearly double the world average of 2.0 kg/ha, underscores significant health and environmental risks, particularly for those in direct contact with these chemicals, as well as for consumers through dietary exposure. The food safety risks are further compounded by poor knowledge of vegetable farmers and their unwillingness to adopt protective measures in handling pesticides and chemicals (Alam & Naser, 2020). Several authors have also reported contamination of vegetables due to (1) the use of inadequately composted manure and improper harvest intervals after manure application (Hok et al., 2020, Ingham et al., 2004); (2) the common practice of cutting out bruised portions of vegetables (Alegbeleye et al., 2022, Camelo, 2004, Hok et al., 2020, Mostafidi et al., 2020, Saranraj et al., 2012); (3) a lack of cold storage (Gorny, 2005, Makule et al., 2022); (4) a lack of hygienic practices during transport and selling (Bekele et al., 2017, Tefera et al., 2014); (5) poor personal hygiene and hand washing habits (Agbalaka et al., 2019); (6) no appropriate training or laws and regulations (Rakha et al., 2022, Shukla et al., 2014); and (7) a lack of adequate tables, roofing, etc. and hygienic infrastructure (functioning drains, cleanable surfaces, etc.) within markets (Hok et al., 2020). This highlights that adopting and implementing food safety measures is not just the responsibility of vegetable farmers but a shared responsibility of all the actors throughout the value chain.

The ability of the value chain actors to ensure vegetables are safe is shaped by their knowledge, attitudes, and practices (KAP) (Wallace et al., 2022). Numerous studies (Al Banna et al., 2021, Haque and Kohda, 2020, Young et al., 2019, Zanin et al., 2017) have evaluated the extent of the actors' knowledge, attitudes, and practices. A general trend based on previous studies, emphasizes that the presence of knowledge, education, and training about food handling abilities has been observed to exhibit a positive correlation with the adoption of good practices. Conversely, it was observed that factors such as education, training, extensive experience in food handling, and attitudes were significantly correlated with an individual's level of food safety knowledge. It is also widely accepted that an individual’s socio-demographic background, knowledge, experiences, and beliefs influence their food safety-related attitudes and practice behavior. Hence, understanding the KAP of vegetable handlers is essential for designing effective interventions to improve food safety. Therefore, this study aims to understand the food safety-related KAP of actors across the vegetable value chain in Bangladesh and identify areas where there are gaps in knowledge and where attitudes may impede the adoption of safe food handling procedures.

## Methods

This was a community-based, cross-sectional study among actors in the vegetable value chain in the Jashore Sadar subdistrict (*upazila*) of Jashore District of Bangladesh. A total of 50 actors (20 retailers, 10 local wholesalers, 10 distant wholesalers and *Aratdars*, and 10 aggregators) were purposively selected for this KAP study. The selection criteria for different actors focused on ensuring that each participant was actively involved in the handling, distribution, or sale of vegetables within the local supply chain and had at least one year of active experience in their respective roles. These roles were chosen because each group provides unique insights into food safety practices within the supply chain, which is essential for a comprehensive assessment. The study was carried out in February–March 2021.

The protocol was approved by the Institutional Ethics Review Board of Bangladesh Agricultural University (approval number: ESRC/35/AERS/2021). Informed consent was obtained orally for all participants. Participants were provided with a detailed explanation of the study's purpose, procedures, potential risks, and benefits. They were assured of the confidentiality of their responses and their right to withdraw from the study at any time without any repercussions. No incentives were provided for participation in the study.

**Figure 1:**
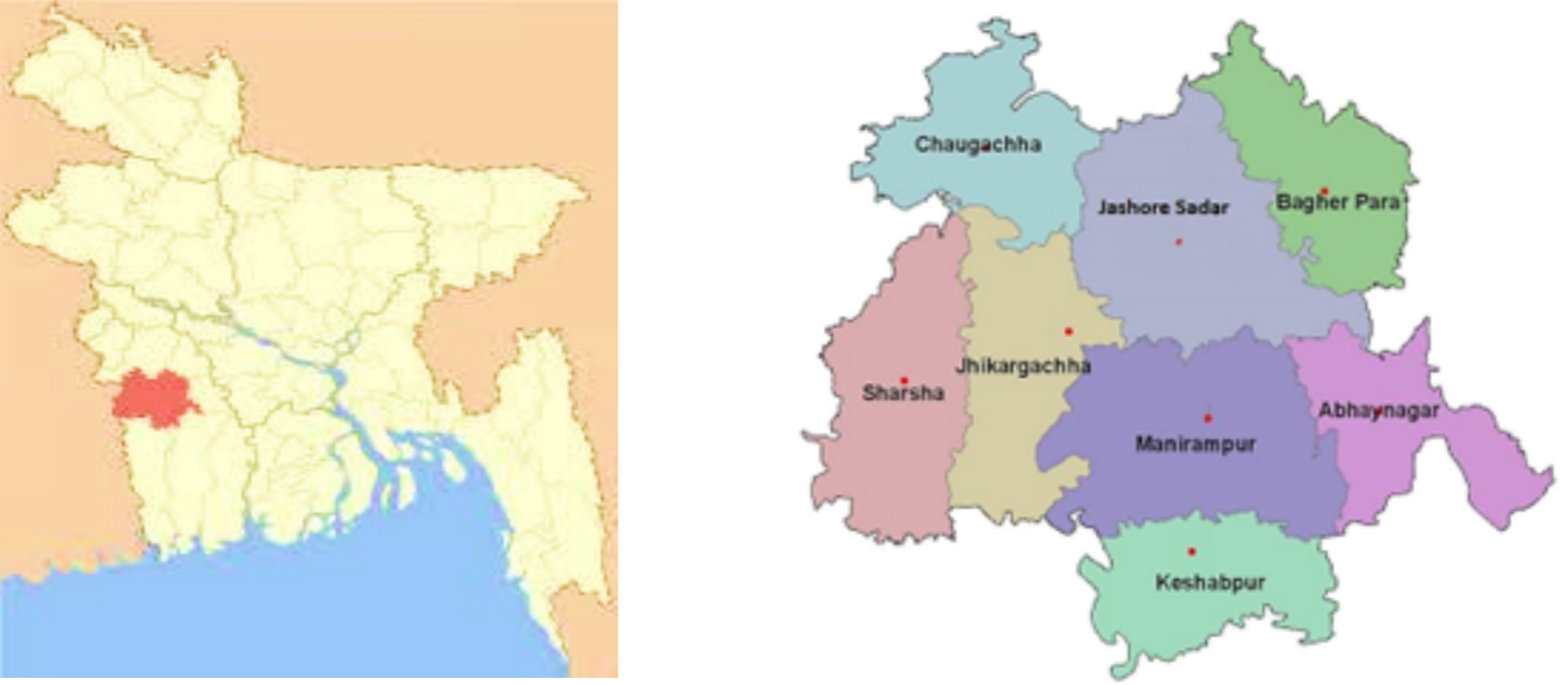
(a) Location of Jashore District (red) within Bangladesh (yellow); (b) Jashore District’s Upazilas. (For interpretation of the references to color in this figure legend, the reader is referred to the web version of this article.)

**Study area.** Jashore Sadar subdistrict (*Upazila*) of Jashore District is located in the northern part of the Khulna division of Bangladesh, approximately 194 km from Dhaka (Fig. 1). Jashore Sadar was selected for this study based on the high vegetable production volumes, 77,199 tons during the 2017–2018 fiscal year (Bangladesh Bureau of Statistics, 2018, Department of Agricultural Extension, 2018). Moreover, the subdistrict is the site of the recent implementation of Digital Green’s (DG) Loop program, which is an aggregation system designed to improve smallholder farmers’ access to remunerative markets. As described by Cooper et al. (2021), a designated aggregator picks up produce from farmers registered with Loop and transports it to the chosen market (in return for a transportation fee determined by the distance of the market and the volume of produce being transported. In turn, this pooling of production enables producers to split transportation costs and utilize motorized vehicles to access larger and further away markets than they might have otherwise through alternative transportation means (e.g., on foot or by bicycle).

**Data collection and analysis.** We used a mixed methods approach consisting of quantitative data collection through concisely formed structured questionnaires and qualitative data collection through focus group discussions (FGDs) to understand food safety-related KAP of vegetable handlers. Value chain actors who directly handled vegetables and provided consent to participate were included in this KAP study.

**Structured questionnaire:** A self-administered questionnaire was used to examine the baseline food safety-related KAP of vegetable handlers. The questionnaire was developed in English and translated into Bangla using standard translation procedures by an expert translator fluent in both languages and familiar with food safety and local practices. This ensured that the content, tone, and intent of each question were accurately conveyed. The translated version was then reviewed by a bilingual expert from our research team, who assessed its cultural and contextual relevance to ensure that terms and phrases were locally appropriate and easily understood by the target population. The questionnaire contained four modules, namely socio-demographics (7 items), knowledge (17 questions), and attitudes. The questionnaire was pretested before the self-administered survey (16 statements) and practices (17 questions). Knowledge, attitudes, and practices were assessed as binary outcomes (yes/no or agree/disagree). We used Bloom’s cut-off points to categorize the overall KAP of the vegetable handlers into three levels: good (80–100%), average (60–79%), and poor (less than 60%) (Azanaw et al., 2021, Barnabas et al., 2024, Rahman et al., 2012).

All the questionnaires were checked visually, coded, and entered into Excel and exported to StataMP 16.0 (StataCorp LLC) for analysis. Descriptive statistics were used to elaborate on the KAP of vegetable handlers and explore the potential relationship between socio-demographic variables and KAP. The internal consistency of the KAP items in the questionnaire was evaluated using Cronbach’s alpha (Alam & Naser, 2020). Cronbach’s alpha values were 0.847 for knowledge, 0.785 for attitudes, and 0.700 for self-reported practices, indicating acceptable reliability of the survey instrument.

**Focus Group Discussions (FGDs):** Five FGDs, each with 10 participants (Table 1), were conducted by the experienced research team composed of three members using an open-ended checklist. The question guide was initially drafted in English and translated into Bangla to facilitate cooperation from the respondents. FGDs were conducted in Bangla to allow for more inclusive participation in the discussion, audio recorded, and supplemented with field notes.

**Table 1:** Distribution of the FGDs

FGDs profile	Location	Number of participants
Loop Aggregators	Satmail Bazaar	10
*Aratdars* and wholesaler	Satmail Bazaar	10
Retailers	Natuapara Bazaar	10
Retailers	Kodaila Bazaar	10
Wholesalers	Lebutala Bazaar	10

Overall, qualitative data collection focused on (1) training on the safe handling of vegetables, (2) knowledge on proper and hygienic handling, transportation, and storage of vegetables, (3) usefulness and application of safe vegetable handling practices, (4) use of chemicals in vegetable handling, and (5) cost implications of adopting proper food safety practices.

The audio recordings were transcribed verbatim, except for repetitive terms or ideas. The transcription was done by the first author, who listened to the recordings and translated them into English. If no exact word or description was available in English, the term in Bangla was used to ensure the capture of all ideas or concepts in translation. The data were coded and thematically analyzed using the statistical software StataMP 16.0 (StataCorp LLC).

## Results

**Socio-demographic characteristics.** The age distribution of the vegetable handlers ranged from 22 to 63 years, with the majority aged 30 years or above. Around half of the people who took the survey have completed some postsecondary education. Most of the handlers reported vegetable trading as their primary business activity (86%), worked in rented premises (56%), and had less than five years of experience (36%). Only 40% of the vegetable handlers had received training on food safety, with nearly one-quarter having received the training more than three years ago (Table 2).

**Table 2:** Vegetables handlers’ socio-demographic characteristics (*n* = 50)

Parameter	Characteristics	Frequency	%
Age (years)	<20	1	2
	20–30	4	8
	30–40	22	44
	>40	23	46

Education	No formal education	6	12
	Primary	16	32
	Secondary	18	36
	College/ university	10	20

Occupation	Farming	7	14
	Business	43	86
	Service	0	0
	Other	0	0

Working space establishment	Own space	22	44
	Rented space	28	56

Working experience (years)	<5	18	36
	5–10	12	24
	10–15	7	14
	>15	13	26

Received training on food safety	Yes	20	40
	No	30	60

If received training	1 = Never attended	30	60
	2 = ≤3 years ago	9	18
	3 = >3 years ago	11	22

**Figure 2:**
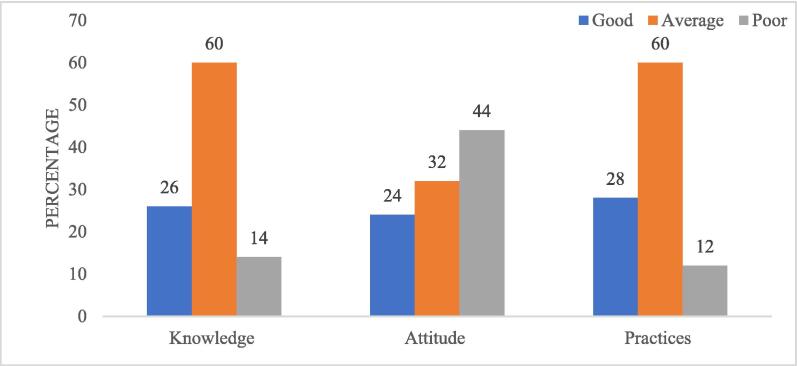
Level of knowledge, attitudes, and practices regarding food safety.

**Food safety-related knowledge, attitudes, and practices among vegetable handlers.** Overall, quantitative data revealed that the majority of the vegetable handlers reported average knowledge (60%) and practices (60%) but a poor attitude (44%) regarding food safety (Fig. 2).

**Knowledge.** The majority of the vegetable handlers demonstrated sufficient knowledge about food safety, safe and hygienic handling, storage of vegetables, risks of vegetable contamination, and effects of unsafe/contaminated vegetables (Table 3). Only two questions, “*Do you know any food safety-related acts or laws of our country?” and “Do you have sufficient knowledge about safe storage practices for vegetables?*” elicited fewer affirmative responses, indicating lower knowledge of these aspects.

**Table 3:** Vegetables handlers’ food safety-related knowledge

No.	Questions on food-safety-related knowledge	%	
		Yes	No
1	Did you ever hear the term “food safety”?	86	14
2	Do you know anything about food poisoning?	86	14
3	Do you have sufficient knowledge about safe handling practices for vegetables?	66	34
4	Do you have sufficient knowledge about safe storage practices for vegetables?	46	54
5	Do you know that microbes (viruses, bacteria, etc.) can contaminate vegetables?	82	18
6	Do you know that microbes can be transmitted from rotten vegetables if mixed with fresh vegetables?	88	12
7	Do you know that microbes can be transmitted to vegetables if kept on the ground or stored in an unclean place?	90	10
8	Do you know that unclean equipment can transmit microbes to vegetables (knives, baskets, containers, etc.)?	82	18
9	Do you know that washing hands before handling vegetables can prevent contamination?	82	18
10	Do you know that using gloves when handling vegetables can prevent contamination?	76	24
11	Do you know that vermin (rats, cockroaches, etc.) and other animals (cats, dogs, cows, etc.) can contaminate the vegetables?	92	8
12	Do you know the harmful effects of using unclean water for washing vegetables?	90	10
13	Do you know the harmful effects of excessive pesticides or insecticides on vegetables?	84	16
14	Do you know the harmful effects of artificial colors or ripening agents on vegetables?	84	16
15	Do you know that unsafe handling practices can deteriorate the quality of vegetables?	82	18
16	Do you know that unsafe food can be the reason for food-borne illnesses (abdominal pain, diarrhoea, vomiting, etc.)?	92	8
17	Do you know any food-safety-related acts or laws of our country?	38	62

**Attitudes.** We examined the attitudes towards food safety through the statements detailed below. As seen in Table 4, most vegetable handlers agreed on the importance and benefits of practicing food safety, personal hygiene, and safe vegetable handling and storage. Nearly all vegetable handlers (94%) reported that “*they were willing to increase their knowledge regarding food safety*,” while 66% stated that “*maintaining food safety practices was too costly*.” Interestingly, only a small percentage of handlers believed that “*assuring food safety was the responsibility of the government*” (18%) or that “*maintaining food safety practices are the responsibility of the consumers*” (24%). In comparison, 42% felt that “*assuring safety is the responsibility of the vegetable farmers*.”

**Table 4:** Vegetables handlers’ food safety-related attitudes

No.	Statement	%	
		Agree	Disagree
1	I do not really understand what food safety is	48	52
2	I think food safety is a complicated issue	54	46
3	I think food safety is not really a business priority in vegetable marketing	42	58
4	I do not have time to maintain safety practices	42	58
5	I cannot see any benefits of maintaining safety practices	30	70
6	I think washing hands before handling vegetables is not necessary	26	74
7	I think using gloves is not necessary to prevent contamination	28	72
8	I do not think that displaying or storing vegetables on the ground can pose any contamination	20	80
9	I do not think that vermin (rats, cockroaches, etc.) and other animals (cats, dogs, cows, etc.) can pose any contamination to vegetables	20	80
10	I think the price is more important than the quality	40	60
11	Maintaining food safety practices is too costly	66	34
12	Maintaining outer product quality (color, shape, spots, etc.) is more important to me	54	46
13	I think assuring safety is the responsibility of the vegetable farmers	42	58
14	I think maintaining food safety practices is the responsibility of the consumers	28	72
15	I think assuring safety is the responsibility of the government	18	82
16	I am willing to increase my knowledge about safety	94	6

**Practices.** Over 90% of the vegetable handlers reported cleaning their workplace and water containers, using clean water for washing vegetables, maintaining clean working space, separating good produce from spoilt vegetables, and practicing good personal hygiene by washing hands after using the toilet or sorting garbage (Table 5). In contrast, only 68% reported “*washing hands after sneezing*,” and an even lower percentage “*washed their hands before touching the vegetables*” (60%) or “*washed their hands after touching money*” (34%). Some handlers also reported “*use of pesticides and chemicals on the vegetables*” (14%) or “*use of color on the vegetables to make them look fresher*” (12%).

**Table 5:** Vegetable handlers’ food safety-related practices

No.	Questions on food safety-related practices	%	
		Yes	No
1	Do you wash your hands before touching the vegetables?	60	40
2	Do you wash your hands after using the toilet?	94	6
3	Do you wash your hands after sneezing?	68	32
4	Do you clean your working place regularly?	90	10
5	Do you wash your hands after sorting the garbage?	96	4
6	Do you wash your hands after touching money?	34	66
7	Do you wear clean clothes while working?	84	16
8	Do you use any baskets to keep the vegetables in, or do you keep them on the ground?	80	20
9	Do you clean the baskets before putting the vegetables in them?	86	14
10	Do you keep the vegetables in direct sunlight?	24	76
11	Do you keep the rotten vegetables mixed with the fresh vegetables?	6	94
12	Do you use clean water to wash the vegetables?	98	2
13	Do you clean the water container regularly?	96	4
14	Do you use any pesticides or chemicals on the vegetables?	14	86
15	Do you use any color on the vegetables to make them look fresher?	12	88
16	Do you allow any animals (cats, dogs, cows, etc.) around the working space?	10	90
17	Do you ask the sellers about their safety practices?	66	34

**Relationship of socio-demographic variables with the KAP of vegetable handlers.** Descriptive statistics were used to explore a possible relationship between the variables and obtain directional insights (Table 6).

**Table 6:** Attribution of KAP relative to educational level, work experience, and training

Construct	Education				Working experience				Have you received training on safety?		When did you receive training?		
	No formal education	Primary	Secondary	College/university	<5 yrs	5–10 yrs	10–15 yrs	>15 ys	Yes	No	Never	≤3 yrs ago	>3 yrs ago
*Knowledge (%)*													
Good	66.7	43.8	66.7	70.0	55.6	75.0	57.1	53.8	85.0	43.3	43.3	88.9	81.8
Average	0	43.8	27.8	10.0	22.2	0.0	0.0	23.1	10.0	36.7	36.7	0.0	18.2
Poor	33.3	12.5	5.6	20.0	22.2	0.0	0.0	23.1	5.0	20.0	20.0	11.1	0.0
*Attitudes (%)*													
Good	33.3	37.5	16.7	10.0	33.3	25.0	0.0	23.1	15.0	30.0	30.0	11.1	18.2
Average	16.7	31.2	61.1	50.0	44.4	50.0	57.1	30.8	50.0	40.0	40.0	44.4	54.5
Poor	50.0	31.2	22.2	40.0	22.2	25.0	42.9	46.2	35.0	30.0	30.0	44.4	27.3
*Practices (%)*													
Good	33.3	25.0	22.2	40.0	33.3	16.7	14.3	38.5	35.0	23.3	23.3	33.3	36.4
Average	50.0	68.8	66.7	40.0	55.6	66.7	85.7	46.2	60.0	60.0	60.0	55.6	63.6
Poor	16.7	6.2	11.2	20.0	11.1	16.7	0.0	15.4	5.00	16.7	16.7	11.1	0.0

Vegetable handlers with 5–10 years of work experience and those who had received training had good food safety-related knowledge. Individuals with higher degrees of education as handlers possessed a good level of knowledge of food safety. Overall, around one-third of the vegetable handlers had a good attitude towards food safety, and interestingly, this was inversely related to their education level, years of work experience, and having received food safety training. Only around 40% of vegetable handlers with a college education and more than 15 years of work experience had good practices related to food safety. While 35% of handlers who received food safety training had good practices, there appeared to be no difference in good practices associated with the timing of training received.

**Qualitative insights on food safety-related KAP among vegetable handlers.** FGDs revealed that most vegetable value chain actors knew about food safety-related aspects and also demonstrated their knowledge in practice. Retailers in the Natuapara group knew how to clean vegetables and store them in a safe manner using clean containers, baskets, and trays. They were aware of the importance of personal hygiene, such as wearing clean clothes and washing hands with soap or hand-wash before touching the vegetables or after using the toilet, using gloves when touching the vegetables (including loading and unloading), and preventing others from touching the vegetables without gloves, or if they had dirty hands. The retailer's group at Kodalia bazaar knew that vegetable containers, baskets, and crates should be washed with clean water, no chemicals or colors should be used, spoiled vegetables should not be mixed with good ones, vegetables should not be put directly on the floor or in contact with soil, and they should be careful regarding insects, rats, and other types of animals. They clarified that they gained this knowledge by watching others and agriculture-related programs on TV and had not received any training on food safety. The local wholesaler group also knew about safe packaging and not keeping vegetables in contact with soil. Some reported having received training, while others obtained this knowledge from the TV, newspapers, and by watching others. Other participants (wholesalers’ group from Lebutala and aggregators) were knowledgeable about safe packaging, transportation, loading/unloading (only wholesalers’ group), grading and sorting (only aggregators), and storage. The wholesalers explained that they gained this knowledge by their own experience and by watching others who maintained the safety of vegetables. All the vegetable handlers indicated that they believed vegetables became unsafe if not properly graded, cleaned, and transported. The correlation between self-reported knowledge and demonstrated actions indicates the validity of the results and emphasizes the practical use of food safety knowledge in the vegetable value chain.

In practice, only the retailer group at Natuapara stated that they washed their hands before handling vegetables, used clean crates to store vegetables, and transported vegetables using *vans* or bicycles. They believed that food safety varied by vehicle type and perceived transportation of their vegetables in vans, together with others’ produce, was the reason for their vegetables getting spoiled. However, as they could not transport/sell as much using a bicycle, they often preferred using a van. The retailer group at Kodalia bazar also reported washing vegetables with clean water and using vans or bicycles for transportation. They preferred using bicycles over vans as they felt it allowed them to ensure safety measures. The retailers reported often using hired laborers for loading, unloading, and washing. They did not use any protective gear (such as gloves) but usually washed their hands with clean water. The distant wholesalers from Lebutala reported using sacks, baskets, and crates to load vegetables. While they believed crates led to fewer losses in produce than sacks, they still used the latter for transporting vegetables as crates were expensive. They mentioned using trucks and trawlers for transporting vegetables and using hired labor for vegetable handling. The aggregators described using *van*s, trucks, pick-ups, and *alamshadhus* to transport vegetables to local wholesale markets. They believed *vans* to be the safest mode of road transport to local wholesale markets and pick-up trucks for distant markets. The aggregators explained that they knew little about the modern technology/ approaches used to handle the vegetables.

Most vegetable handlers clarified that they regularly cleaned their vegetable containers and weighing machines. They understood that keeping containers and weighing machines clean was essential, stating, for example, that “*we know that unsafe handling practices can deteriorate the quality of vegetables.*” Local wholesalers reported that they cleaned containers regularly while weighing machines were cleaned once a week or fortnightly using clean water and cloth. Distant wholesalers from Lebutala informed us that they did not wash their containers or weighing machines daily. Aggregators also highlighted using clean water and cloth to clean the crates, sacks, baskets, and weighing machines.

All the vegetable handlers, except for the retailers at Kodalia Bazzar, graded their vegetables according to quality, size, and appearance. They also explained that they would discard the grade-C or *kat* vegetables (considered poor quality or spoilt) and sell the grade-A or *good* and grade-B or *poka* in the market. Retailers from Kodalia bazaar stated they only bought good quality vegetables and sold them to customers. The groups mentioned that the price difference between grade-A and grade-B vegetables was around BDT 5–6 (USD 0.046–0.055) per kg.

All groups of vegetable handlers were aware of the adverse health impacts caused by unsafe food products as well as of the short- and long-term consequences of unsafe vegetable handling practices. However, the majority of the groups also felt that adopting food safety practices would increase their costs. The Kodalia bazaar retailers and local wholesalers opined that the requirements for purchasing soap, protective gear, etc., would increase their costs. Furthermore, they believed that extra labor would be needed for grading and sorting, which would further increase costs by around BDT 200 (USD 1.82)/maund. The wholesalers from Lebutala speculated on a cost increase of BDT 40 (USD 0.36)/maund, while the aggregators asserted that this would lead to an average cost increase of BDT 100 (USD 0.91)/maund. In contrast, the Natuapara retailer group felt that there might be a slight decrease in costs from not using chemicals or coloring agents, but only if all the other actors followed the practice.

As proper handling ensures safety standards, all groups of vegetable handlers agreed that following adequate safety practices would increase the prices for vegetables by BDT 20–40 (USD (0.18–0.36)/maund. However, they also believed that the price premium would only cover some of the additional costs incurred and were concerned about all actors not adopting the safety measures. They believed that the government should have laws to enforce food safety practices among the various vegetable handlers.

When enquired about a possible economic benefit to them, all the groups stated that adhering to proper practices would increase their income. Local wholesaler groups believed they could increase their sales by BDT 100–150 (USD 0.91–1.37)/maund of vegetables. They believed that adequate and safe handling of vegetables would reduce wastage and attract consumers, thereby increasing their sales and profit.

## Discussion

Proper vegetable handling plays a significant role in ensuring its quality and safety for consumption (Kader & Rolle, 2004). Up-to-date knowledge of the vegetable handling practices across the value chain is essential to design preventive measures for mitigating contamination of vegetables and, thereby, their contribution to foodborne diseases. This research delves into food safety-related KAP among vegetable handlers in Jashore Sadar in Bangladesh to gain pivotal insights that can help ensure the safety and quality of vegetables reach consumers' plates. We employed an innovative mixed-method research design to investigate the KAP of various actors across the vegetable value chain for a comprehensive understanding of the determinants of the safe handling of vegetables in the food supply chain. Our research revealed a mixed landscape regarding the KAP of vegetable handlers in the context of food safety in Jashore Sadar. While individuals involved in handling vegetables exhibited a moderate level of knowledge and adherence to food safety practices, a clear need to improve positive attitudes toward food safety was noted.

Prior research (Abdul-Mutalib et al., 2012, Al-Kandari et al., 2019, Gemeda et al., 2023, Van Boxstael et al., 2013, Wertheim-Heck et al., 2014) emphasized the importance of knowledge in ensuring food safety. At the same time, specific gaps in knowledge, particularly related to food safety-related laws and safe storage practices, were also noted and are in line with earlier studies (Faour-Klingbeil, 2022, Hoffmann et al., 2019, Lamm et al., 2021, Prabhakar et al., 2010). Notably, a substantial proportion of vegetable handlers in our study exhibited sufficient knowledge about food safety principles and safe handling. The exemplary baseline of knowledge among these handlers may be attributed to the favorable influence of sufficient exposure to food safety, quality, and standards concerns by local authorities, awareness campaigns, and informal learning within the community (Ko, 2010). The majority of the handlers in this study acknowledged the importance and benefits of food safety practices but also expressed concerns about the costs involved. Specific barriers such as the cost of gloves, cleaning agents, and lack of affordable training contributed to handlers’ reluctance toward consistent safety practices. Different roles along the supply chain reported varying cost burdens, underscoring the need for subsidized resources or policy interventions to offset these expenses. These attitudinal findings align with those of Larson (2021), who noted that perceived cost can influence compliance with food safety measures. A substantial number of handlers attributed the responsibility for ensuring food safety to vegetable farmers, which may indicate a need for broader education and awareness campaigns (Marine et al., 2016, Parker et al., 2012, Van Boxstael et al., 2013).

Consistent with prior research, this study found a moderate level of hygiene and safe handling practices among vegetable handlers (El-Nemr et al., 2019, Galgamuwa et al., 2016, George et al., 2021). However, hand hygiene practices such as handwashing after sneezing or touching money warrant improvement, as inadequate hand hygiene can pose a significant risk to food safety (Gil et al., 2015, Salamandane et al., 2020). The reported excessive use of pesticides, chemicals, and color on vegetables is also a matter of concern and requires regulatory attention to ensure consumer safety (Din et al., 2011, Gil et al., 2015, Islam and Hoque, 2013, Mostafidi et al., 2020).

The correlation between attitudes towards food safety and knowledge and practices presents challenges in discerning the distinction between attitudes and practice. People’s attitudes are strongly correlated to their knowledge and practices on one hand and to their awareness on the other. It is, therefore, not easy to distinguish attitudes from practice (Zanin et al., 2017). The consensus among handlers was that the practice of food safety, personal hygiene, and safe vegetable handling and storage is of utmost importance (Gil et al., 2015, Insfran-Rivarola et al., 2020, Ko, 2010, Parikh et al., 2022).

Enhancing food safety protocols throughout the entire process of producing and distributing vegetables is essential to minimize the economic damages caused by foodborne diseases, enhance consumer confidence in the food system, and impact their food safety behavior (Lagerkvist et al., 2018). Vegetable handlers exhibited an awareness of the advantageous economic outcomes stemming from implementing food safety measures. Their recognition of the potential benefits, including heightened sales, minimized wastage, and increased consumer trust, underscores the prospect of mutually beneficial results for stakeholders across the entire supply chain. This collective acknowledgment emphasizes the economic synergy achievable through the conscientious practice of food safety measures, reflecting a shared interest in fostering positive outcomes and sustainability throughout the vegetable supply chain (Rajapaksha, Gunathilake, & Pathirana, 2021, Uyttendaele et al., 2015).

The strengths of this study lie in its comprehensive assessment of KAP among vegetable handlers in the context of Bangladesh. The combination of quantitative and qualitative data provides a nuanced understanding of their behaviors and beliefs. The mixed-methods approach allows for insights from different perspectives and enhances the study's credibility. The unexpected finding of this study lies in the detailed knowledge and practices of vegetable handlers, which, despite some gaps in attitudes, largely favor food safety. The qualitative insights from focus group discussions reveal a general awareness of best practices. The novelty of this study lies in its focus on an often-overlooked segment of the food supply chain, on the intricacies of KAP among vegetable handlers. Nonetheless, certain limitations should be acknowledged. The study's cross-sectional design does not allow for causal inferences. The findings are also limited to the specific geographical context and may need to be generalizable to other regions. Additionally, as with self-reported data, there may be a degree of reporting bias regarding practices. It should also be noted that this study did not collect detailed information regarding the specific cleaning agents used or standardized definitions of ‘clean’. While this study captured general cleaning practices, future research should investigate specific parameters and cleaning agents to offer a more comprehensive understanding of hygiene practices.

Our study unraveled specific knowledge gaps, indicating areas where targeted interventions are essential to fortify food safety within the food supply chain. There is a need for future studies to explore the factors influencing vegetable handlers' attitudes and practices, the effectiveness of educational interventions, and the economic implications of adopting food safety measures. It is evident that educational campaigns addressing specific knowledge gaps, particularly regarding food safety-related laws and storage practices, are necessary. These efforts should emphasize the economic benefits of adhering to food safety practices, such as reduced losses and increased sales, to alleviate concerns about increased costs. Additionally, regulatory measures to monitor the use of pesticides, chemicals, and color on vegetables are warranted to safeguard consumer health. Investigations in other regions and an examination of the broader implications of food safety within the vegetable supply chain could provide a more comprehensive understanding of this critical issue.

This study underscores the need to enhance food safety knowledge and practices among vegetable handlers. It offers insights that can drive positive changes in the industry and, ensure the safety of vegetables for consumers.

## Conclusion

This research tried to offer significant contributions towards food safety by shedding light on the KAP exhibited by those involved in handling vegetables throughout the value chain in Jashore Sadar, Bangladesh. The results indicate that a significant proportion of vegetable handlers had a good comprehension of food safety principles, vegetable handling techniques, storage practices, and the potential hazards linked to vegetable contamination. However, practices and attitudes toward personal hygiene and the safe handling of vegetables warrant attention. The poor attitudes and insufficient practices may be due to concerns regarding the potential rise in expenses related to the implementation of food safety measures. However, most vegetable handlers also believed adhering to these practices would ultimately result in enhanced earnings. This would be achieved through the reduction of losses, increased sales, and the ability to charge higher prices for vegetables that are deemed safe. This study also identified significant knowledge gaps related to food safety regulations, laws, and safe storage protocols, highlighting a potential for targeted educational and training programs.

In light of the fact that most of the vegetables in the subcontinent are traditionally cooked before consumption, it is advisable that forthcoming studies investigate how these cooking methods affect the attitudes of vegetable handlers toward food safety. Such an inquiry could yield a more comprehensive understanding of how regional consumption practices influence attitudes and behaviors concerning the microbial food safety of vegetables. Another suggestion for future studies is to include clear definitions of standardized terms to enhance comparability with food safety standards.

## Ethical statement

This study was approved by the Research Ethics Committee of Bangladesh Agricultural University, Mymensingh, Bangladesh (ESRC/35/AERS/2021) on January 15, 2021. Written informed consent was obtained from all the participants. The questionnaires were anonymized, and respondents were free to opt out of participation in the survey.

## Financial disclosure

The authors gratefully acknowledge financial support from the Market Intervention for Nutritional Improvement (MINI) project funded by the 10.13039/100000865Bill & Melinda Gates Foundation (BMGF) and the UK Government’s Foreign, Commonwealth and Development Office (FCDO) (Grant No. OPP1182694). The views expressed in this work are those of the creators and do not necessarily represent those of BMGF, FCDO, or Digital Green.

## CRediT authorship contribution statement

**Ismat Ara Begum:** Writing – original draft, Methodology, Investigation, Formal analysis, Conceptualization. **Mohammad Jahangir Alam:** Writing – review & editing, Methodology, Investigation, Formal analysis, Conceptualization. **Bhavani Shankar:** Writing – review & editing, Validation, Supervision, Resources, Conceptualization. **Tamanna Mastura:** Writing – review & editing, Investigation, Formal analysis, Data curation. **Gregory Cooper:** Writing – review & editing, Visualization, Validation, Methodology, Conceptualization. **Karl Rich:** Writing – review & editing, Visualization, Validation, Methodology, Investigation, Conceptualization. **Panam Parikh:** Writing – review & editing, Visualization, Validation. **Nazmun N. Ratna:** Writing – review & editing, Visualization, Validation. **Suneetha Kadiyala:** Writing – review & editing, Validation, Methodology, Conceptualization.

## Declaration of competing interest

The authors declare that they have no known competing financial interests or personal relationships that could have appeared to influence the work reported in this paper.
